# Vpx is Critical for SIVmne infection of pigtail macaques

**DOI:** 10.1186/1742-4690-9-32

**Published:** 2012-04-24

**Authors:** Michael Belshan, Jason T Kimata, Charles Brown, Xiaogang Cheng, Anna McCulley, Alison Larsen, Rajesh Thippeshappa, Vida Hodara, Luis Giavedoni, Vanessa Hirsch, Lee Ratner

**Affiliations:** 1 Department of Medical Microbiology and Immunology, Creighton University, Omaha, NE, USA; 2 Department of Molecular Virology and Microbiology, Baylor College of Medicine, Houston, TX, USA; 3 Laboratory of Molecular Microbiology, National Institute of Allergy and Infectious Diseases, Bethesda, MD, USA; 4 Division of Molecular Oncology, Washington University School of Medicine, St Louis, MO, USA; 5 Department of Virology and Immunology, Southwest National Primate Research Center, Texas Biomedical Research Institute, San Antonio, TX, USA; 6 Division of Oncology, Washington University, Box 8069, 660 S Euclid Ave, St Louis, MO 63110, USA

**Keywords:** Vpx, SIV, Macaques

## Abstract

**Background:**

Viral protein X (Vpx) of SIV has been reported to be important for establishing infection *in vivo*. Vpx has several different activities *in vitro*, promoting preintegration complex import into the nucleus in quiescent lymphocytes and overcoming a block in reverse transcription in macrophages. Vpx interacts with the DDB1-CUL4-DCAF1 E3 ligase complex, which may or may not be required for the ascribed functions. The goal of the current study was to determine whether these activities of Vpx are important *in vivo*.

**Results:**

An infectious, pathogenic clone of SIVmne was used to examine correlations between Vpx functions *in vitro *and *in vivo*. Three previously described HIV-2 Vpx mutants that were shown to be important for nuclear import of the preintegration complex in quiescent lymphocytes were constructed in SIVmne: A *vpx*-deleted virus, a truncation of Vpx at amino acid 102 that deletes the C-terminal proline-rich domain (X(102)), and a mutant with tyrosines 66, 69, and 71 changed to alanine (X(y-a)). All mutant viruses replicated similarly to wild type SIVmne027 in primary pigtail macaque PBMCs, and were only slightly retarded in CEMx174 cells. However, all the *vpx *mutant viruses were defective for replication in both human and pigtail monocyte-derived macrophages. PCR assays demonstrated that the efficiency of reverse transcription and the levels of viral integration in macrophages were substantially reduced for the *vpx *mutant viruses. *In vitro*, the X(y-a) mutant, but not the X(102) mutant lost interaction with DCAF1. The wild type SIVmne027 and the three *vpx *mutant SIVs were inoculated by the intra-rectal route into pigtail macaques. Peak levels of plasma viremia of the *vpx *mutant SIVs were variable, but consistently lower than that observed in macaques infected with wild type SIVmne. *In situ *hybridization for SIV demonstrated that compared to wild type SIVmne infected macaques five of the six animals inoculated with the *vpx *mutant SIVs had only low levels of SIV-expressing cells in the rectum, most intestinal epithelial tissues, spleen, and mesenteric and peripheral nodes.

**Conclusions:**

This work demonstrates that the activities of Vpx to overcome restrictions in culture *in vitro *are also likely to be important for establishment of infection *in vivo *and suggest that both the nuclear localization and DCAF1-interaction functions of Vpx are critical *in vivo*.

## Background

The *vpx *and *vpr *genes of HIV-2 and SIVmac evolved from a common ancestral gene product as a result of non-homologous recombination [1-3]. Vpx is a protein with 112 residues, that is predicted to have 3 alpha helices and a proline-rich tail [4-7]. It is efficiently packaged into virions [8]. Although Vpx is dispensable for infection of proliferating cells, it is critical for infection of quiescent cells. Early studies established a role of Vpx in the nuclear import of preintegration complexes in quiescent cells [9,10]. The PIC nuclear import activity is mediated by two distinct Vpx domains that include tyrosine-rich sequences within residues 65-72 in the third predicted alpha helix of Vpx and a proline-rich domain in residues 102-112C-terminal to the predicted alpha helical core of the protein [10-12]. Interactive proteins that promote Vpx nuclear import activity include α-actinin, importins α and β, nucleoporins, and heat shock protein 40 member DnaJB6 [13-16].

More recent studies demonstrate that Vpx promotes reverse transcription in macrophages and dendritic cells [17-19]. The activity in macrophages has been attributed to Vpx binding to a ubiquitin ligase complex containing cullin4a (CUL4), damaged DNA-binding protein 1 (DDB1), and the DDB1- and CUL4-associated factor 1 (DCAF1) [18-21]. Vpx residue Q76 and Vpx ubiquitination have been shown to be important for interaction with DCAF1 [18-21]. The target of ubiquitination, that restricts reverse transcription by this complex in the absence of Vpx, has recently been ascribed to SAM-domain HD-domain containing protein 1 (SAMHD1), a product of an unconventional cell-intrinsic innate immune response [22,23]. SAMHD1 appears to function as a deoxynucleoside triphosphate triphosphohydrolase [24,25]. It is unclear which of these distinct activities of Vpx in quiescent cells is dominant, and whether these activities are cell-type dependent.

Vpx has also been shown to be important for the ability of the acutely pathogenic variant of SIVsmm pbj to establish infection *in vivo *[26]. After intrarectal inoculation, both wild type and Vpx mutant SIVs were transmitted across the rectal mucosa and detected by *in situ *RNA hybridization and immunohistochemistry within 4 days of inoculation. More than ninety percent of cells infected with either virus in the rectal mucosa were lymphocytes. Macrophages represented a minority of the infected cell population. Local amplification of infection and dissemination was observed with wild-type SIV, but dissemination of SIVsmm pbj ΔVpx was retarded. Slower dissemination of SIVsmm pbj ΔVpx compared to wild type virus was also seen after intravenous inoculation. In order to confirm and extend these findings, the current study was carried out to investigate the in vivo relevance of specific functions ascribed to Vpx *in vitro *and *in vivo *using SIVmne virus, a less acutely pathogenic and more AIDS-like model of HIV infection than SIVsmm pbj [27].

## Results

### Characterization of SIVmne Vpx mutations

**Figure 1:**
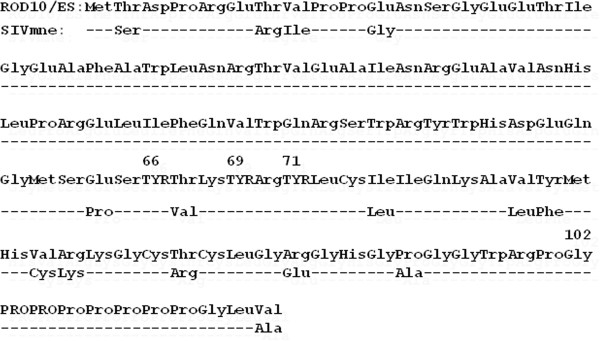
**Sequence alignment of HIV-2rod and SIVmne Vpx**. The amino acid sequence of HIV-2 strain ROD10/ES Vpx is provided with differences present in SIVmne indicated, and dashes representing residues identical in both Vpx products. Tyrosine residues subjected to mutation in SIV-X(y-a) are underlined, and their codon numbers are indicated above each residue. Pro residues 103-104 are underlined since they were substituted with termination codons in SIV-X(102), with the C-terminal Gly residue in the predicted truncated protein indicated by the number above the residue.

**Figure 2:**
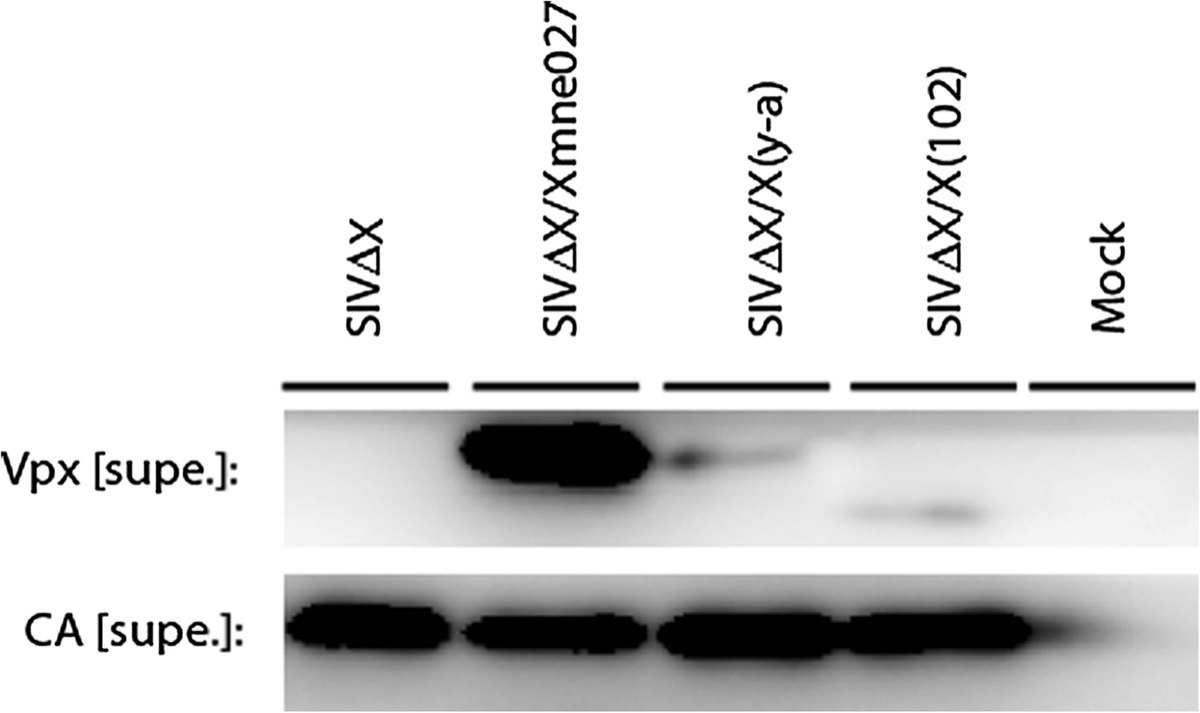
**Expression and packaging of SIV Vpx mutant molecular clones**. SIV molecular clones lacking Vpx expression were complemented *in trans *with indicated Vpx mutants and Vpx WT by transient transfection of 293 T cells. MG132 was added 24 h post-transfection. Supernatants were collected at 48 h and virions were concentrated by ultracentrifugation through a 20% sucrose cushion. Vpx and CA expression were analyzed by immunoblot.

Previous studies of HIV-2rod Vpx demonstrated a critical role for nuclear localization of two domains encompassing a tyrosine-rich sequence within residues 65-72 and a proline-rich sequence within residues 101-112 (Figure 1) [10,11]. In order to determine the role of these domains *in vivo*, mutations were introduced into a pathogenic molecular clone, SIVmne027 [27-29]. Overall, SIVmne Vpx differs from HIV-2rod Vpx at 15 codons (13.4%), situated in the predicted 3rd α-helix and N- and C-terminal to the helical protein core [7]. The first clone, SIVΔVpx, contained mutations that eliminated the initiator methionine codon and introduced a termination codon in place of residue 20 in order to eliminate Vpx expression. The second mutant, SIV-X(y-a), included substitutions of tyrosines 66, 69, and 71 with alanines. The third mutant, SIV-X(102), introduced two termination codons in place of proline residues 103-104, resulting in a truncated protein that deleted the poly-proline stretch at the C-terminus of the protein. The expression and packaging of each Vpx mutant protein was examined by immunoblotting (Figure 2 and Additional file 1: Figure S1). In previous studies of HIV-2rod, both mutants were expressed and incorporated into virions [11]. With SIVmne027, lower band intensities compared to wild type were seen with the mutants, which could reflect differences in stability or packaging.

**Figure 3:**
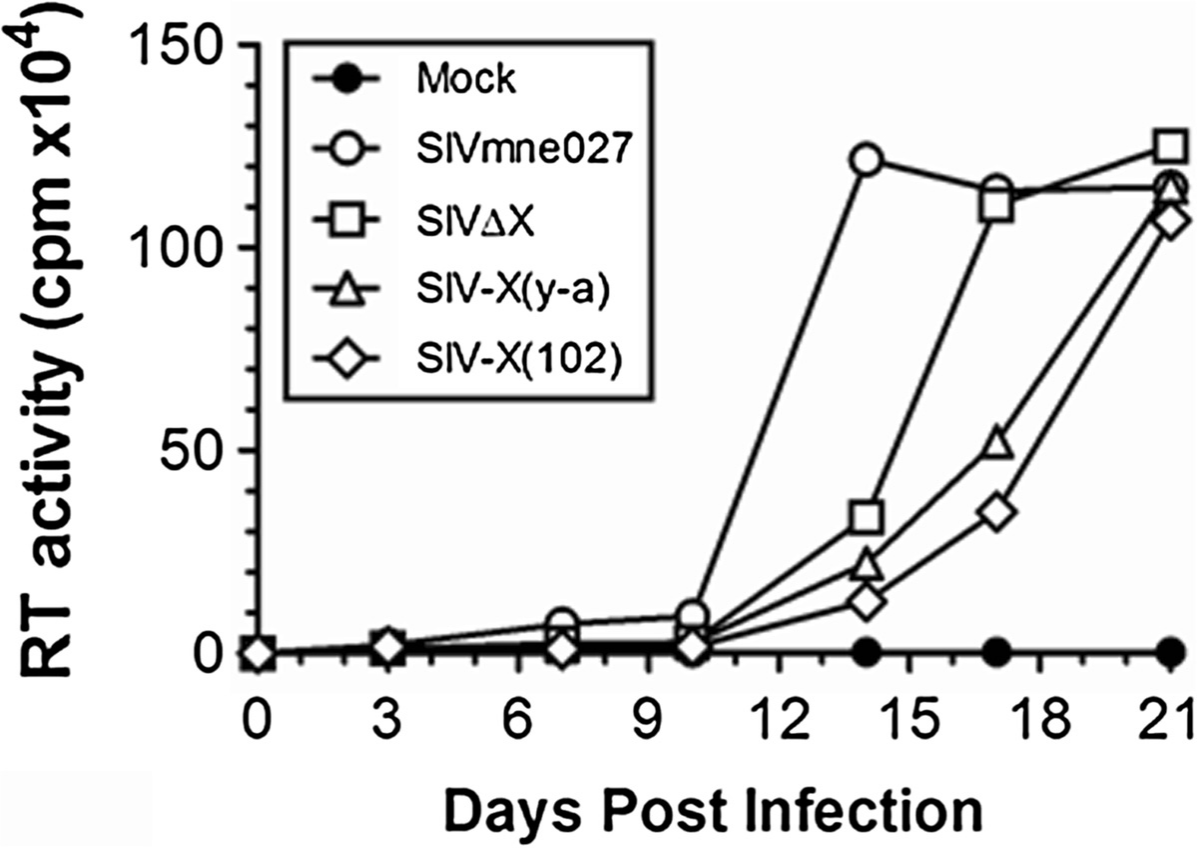
**Replication fitness of SIV Vpx mutant molecular clones in CEMx174 cells**. Wild type and Vpx mutant SIVs (50 ng by p27 antigen ELISA) were used for infection, and reverse transcriptase activity in solubilized released virus present in 0.08 ml of culture supernatant was measured as cpm of [^3^H]-thymidine incorporation into DNA at each indicated time point.

**Figure 4:**
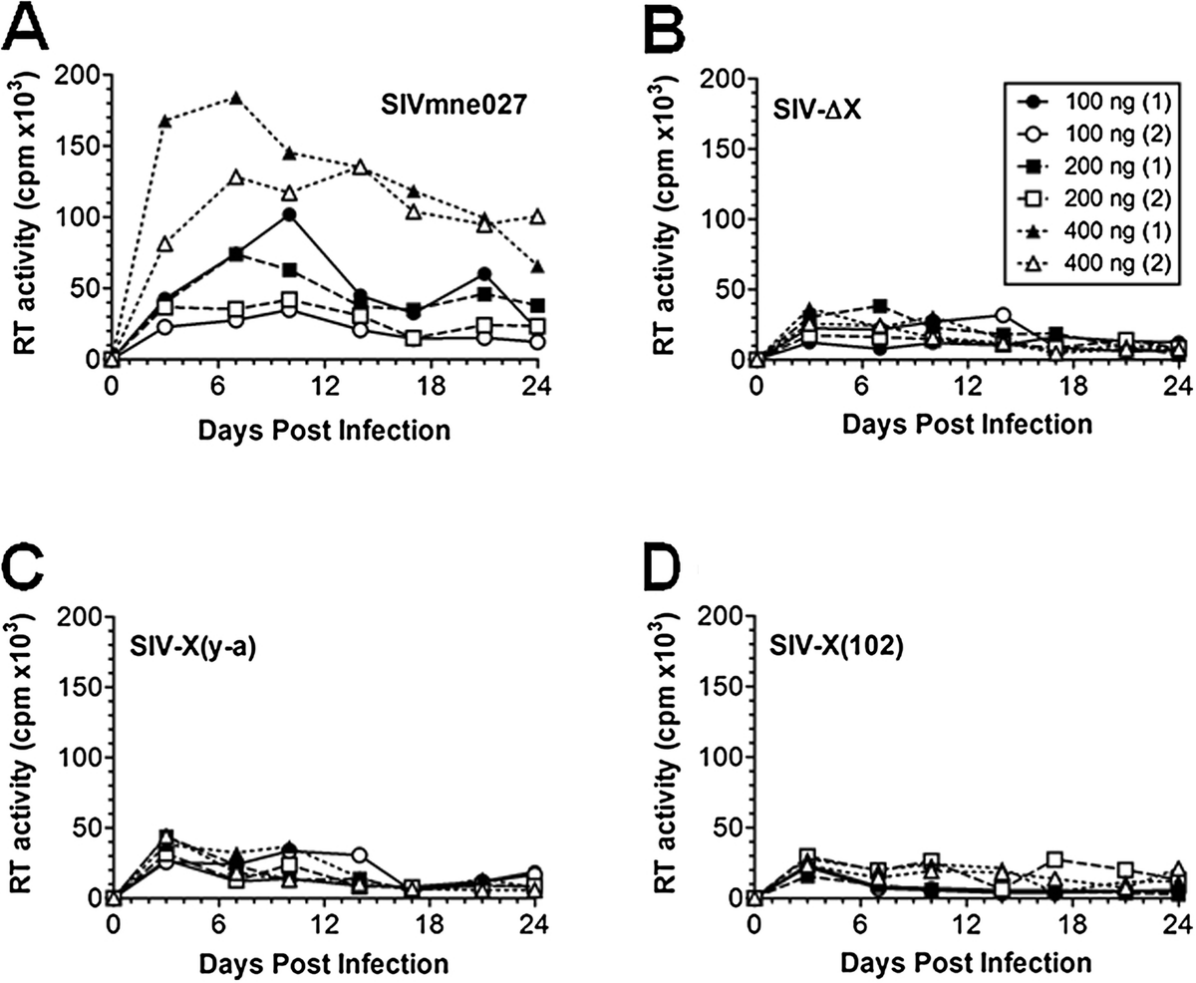
**Replication of SIV Vpx mutant molecular clones in primary human macrophages**. **A) **Wild type and **B-D) **Vpx mutant SIVs, in the amounts indicated in the key and performed in duplicate experiments, were used to infect M-CSF-stimulated human monocyte-derived macrophages, and reverse transcriptase activity was measured, as described in Figure 2.

The replication fitness of the SIVmne-derived molecular clones was assessed in both cell lines and primary cells. First, virus replication was measured in CEMx174 cells (Figure 3). Cells were infected with equivalent amounts of each virus, and virus production assessed in culture supernatants by reverse transcriptase activity measurements. All four viruses replicated to high levels, although the mutant Vpx SIVs were slightly retarded in their replication kinetics compared to wild-type SIVmne027. Next, the replication of the mutant Vpx SIVs was examined in primary human monocyte-derived macrophages (MDMs), using 100-400 ng of each virus, based on p27 antigen ELISA, and monitored by reverse transcriptase activity measurements (Figure 4). Only SIVmne027 gave rise to high levels of replication in MDMs during the 24 days of culture, with average peak levels of reverse transcriptase activity of 68,000, 58,000, and 160,000 cpm/0.08 ml in cultures initiated with 100, 200, or 400 ng of virus, respectively. In contrast, SIVΔVpx, SIV-X(y-a), and SIV-X(102) infection of MDMs gave rise to average peak reverse transcriptase levels < 42,000 cpm/0.08 ml at all dilutions.

**Figure 5:**
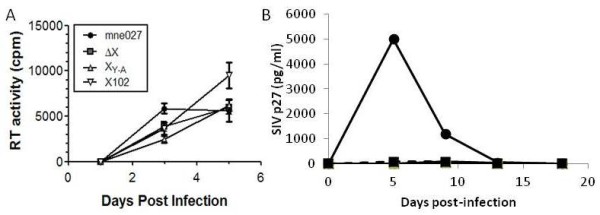
**Replication of SIV Vpx mutant molecular clones in primary macaques PBMCs and macrophages**. **A) **Wild type and Vpx mutant SIVs were used to infect macaque PBMCs, and reverse transcriptase activity measured, as described in Figure 2, or **B) **M-CSF-stimulated macaque monocyte-derived macrophages, and SIV p27 antigen was measured.

Finally, the replication of the mutant Vpx SIVs was examined in primary macaque peripheral blood mononuclear cells (PBMCs) and MDMs (Figure 5). SIVmne027 and each of the mutant SIV variants replicated to similar levels in PBMCs (Figure 5A). Similar to the experiments with human MDM, only SIVmne027 was capable of productive infection in macaque MDMs; none of the mutant Vpx SIV viruses produced detectable levels of p27 Gag antigen in the MDM infections (Figure 5B).

**Figure 6:**
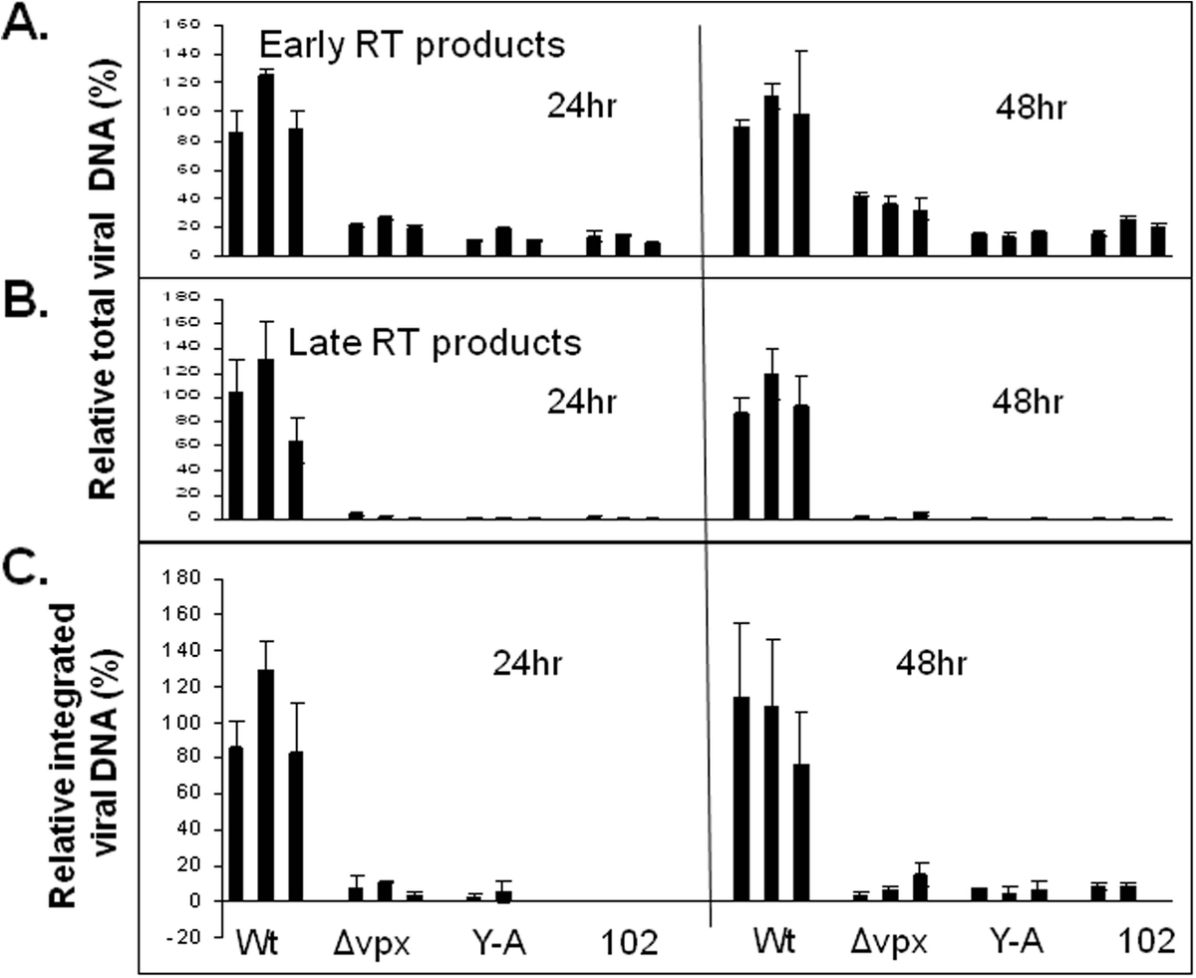
**Synthesis and integration of SIV DNA in primary human macrophages**. Wild type and Vpx mutant SIVs were pseudotyped with VSV-G and used to infect M-CSF-stimulated human monocyte-derived macrophages. After 24 or 48 hrs, total cellular DNA was harvested and subjected to PCR reactions for **A) **early or **B) **late reverse transcription products, or **C) **levels of integrated viral DNA, performed in triplicate assays. Levels of viral DNAs are relative to the average obtained with wild type SIVmne at each time point. The bars in each set of assays indicate standard deviations of assays.

To determine if the failure of the Vpx mutants to replicate in MDMs resulted from an inhibition of reverse transcription, the early events of virus infection were characterized by real time PCR analysis. Primary human MDMs were infected with VSV-G pseudotyped SIVs in triplicate. Viral DNA was extracted at 24 and 48 h post-infection for PCR analysis (Figure 6). Consistent with the finding that Vpx is required for efficient reverse transcription in macrophages, both early and late viral DNA products were substantially reduced in the mutant Vpx SIV infected cells. At 24 and 48 h, the levels of early reverse transcription products in cells infected with mutant SIV viruses were reduced to 10-20% and 15-40%, respectively, of the levels seen in the cells infected with wild type SIVmne027 (Figure 6A). The levels of late reverse transcription products (Figure 6B) and integrated (Figure 6C) viral DNA sequences in macrophages infected with mutant viruses were also reduced to < 5% and < 10%, respectively, of the cells infected with wild type SIVmne027. These findings are consistent with the previously reported role of Vpx in promoting reverse transcription in macrophages or promoting the stability of the reverse transcriptase products [18,19]. These findings also demonstrate that both the triple tyrosine and C-terminal proline domains are necessary for this function of Vpx.

**Figure 7:**
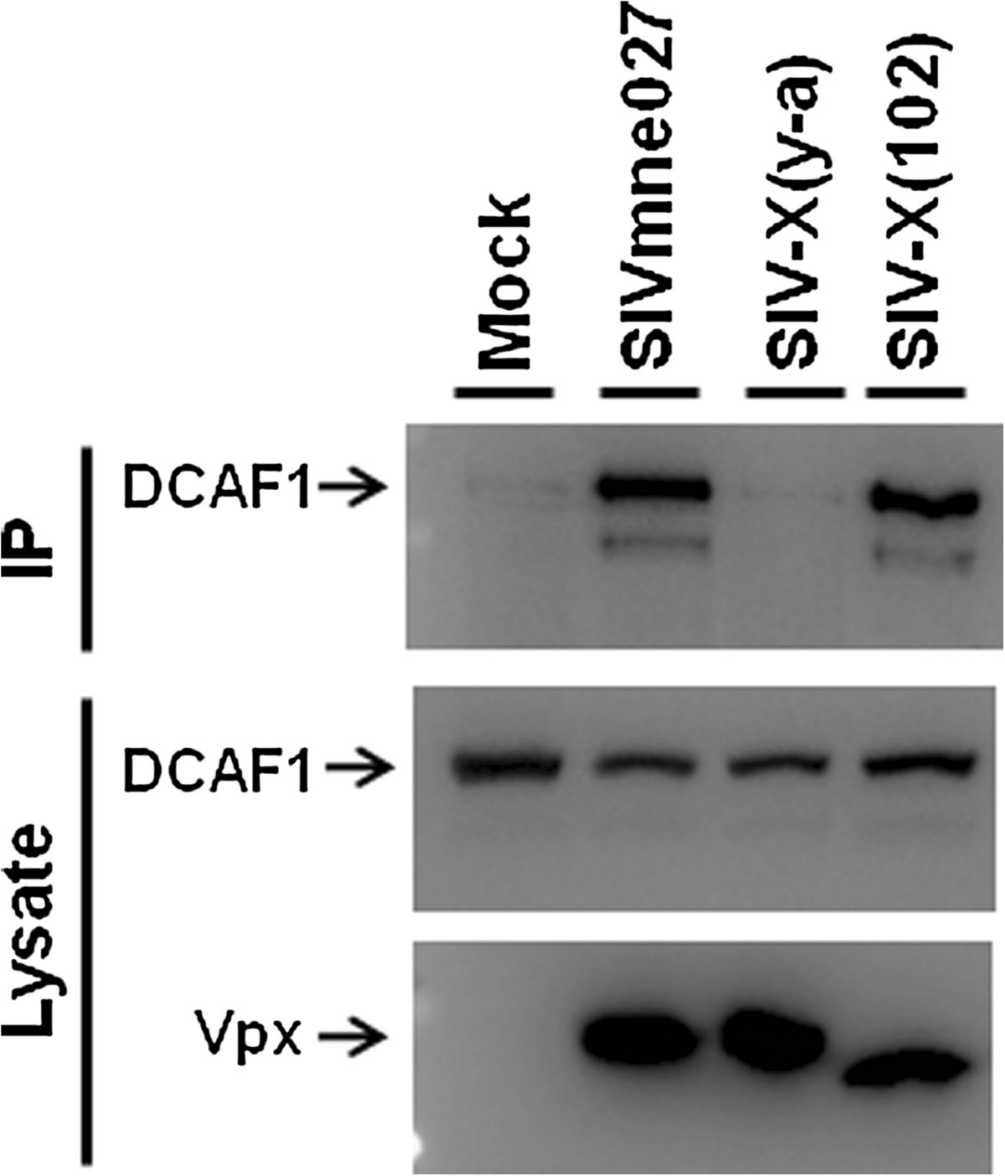
**Interaction of SIV Vpx and X-102 with DCAF1**. FLAG-tagged wild type and mutant SIV Vpx expression clones were transfected into 293 T cells, which were treated with 5 μM MG-132 at 24 h post-transfection. After 48 hrs post-transfection cell lysates were immunoprecipitated with anti-FLAG antibody, and analyzed on SDS-PAGE and immunoblot with anti-DCAF1 antibody (top panel). Total cell lysates were analyzed by SDS-PAGE and immunoblot with anti-DCAF1 and anti-FLAG (bottom panels).

Since the activity in macrophages has been attributed to Vpx binding to a ubiquitin ligase complex containing DCAF1, we analyzed the SIV Vpx mutants for their ability to bind DCAF1 (Figure 7) [18-21]. For this purpose, a FLAG epitope tagged version wild type SIV Vpx and each mutant was expressed in 293T cells. Interactions with endogenous DCAF1 were analyzed by immunoblot with a DCAF1 monoclonal antibody using the anti-FLAG immunoprecipitates.(top panel). In contrast to wild type Vpx and the SIV-X(102) mutant, the SIV-X(Y-A) mutant did not bind to DCAF1, suggesting that Vpx interactions with factors in addition to DCAF1 may be important for the ability of Vpx to promote reverse transcription in these cells.

**Figure 8:**
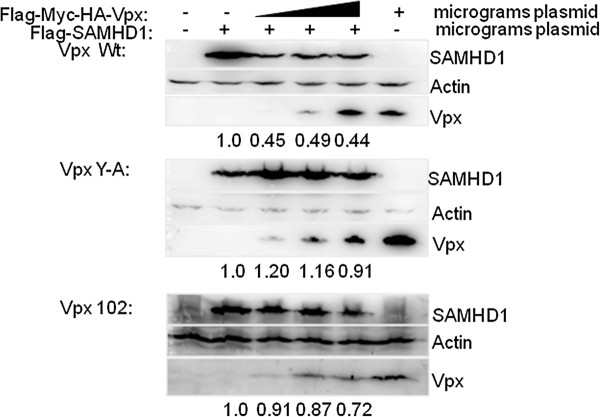
**Degradation of SAMHD1 by SIV Vpx and X-102**. Increasing amounts of FLAG-tagged wild type and mutant SIV Vpx expression clones were co-transfected with FLAG-tagged SAMHD1 expression clones into 293T cells. After 72 hrs cell lysates were analyzed on PAGE-SDS and immunoblotted with anti-Flag and anti-Actin antibodies. Positions of SAMHD1, Actin, and Vpx in the blots are indicated. Densitometry values (normalized to levels of actin), relative to SAMHD1 in the absence of Vpx, are shown below each blot.

Since recent studies demonstrated that SAMHD1 is a cellular restriction factor for HIVs and SIVs in myeloid cells, whose stability is regulated by DCAF1 and Vpx [22,23], we assessed the stability of FLAG-tagged SAMHD1 in the presence of wild type and mutant SIV Vpx proteins (Figure 8). Co-expression of increasing amounts of wild type Vpx resulted in 51-56% decreased levels of SAMHD1 in 293T cells 72 hrs after transfection (Figure 8, top panel). Co-expression of increasing amounts of SIV-X(102) mutants resulted in 9-28% decreased levels of SAMHD1 (Figure 8, bottom panel), which was significantly different from the results seen with wild type Vpx, (p < 0.01). Co-expression of increasing amounts of SIV- X(Y-A) had little if any effect on levels of SamHD1 (Figure 8, middle panel), which was also significantly different from the results seen with wild type Vpx (p < 0.01). The effects on SAMHD1 stability of SIV-X(102) and SIV-X(Y-A) were not significantly different (p = .055).

### Replication of SIVmne Vpx mutants in pigtail macaques

**Figure 9:**
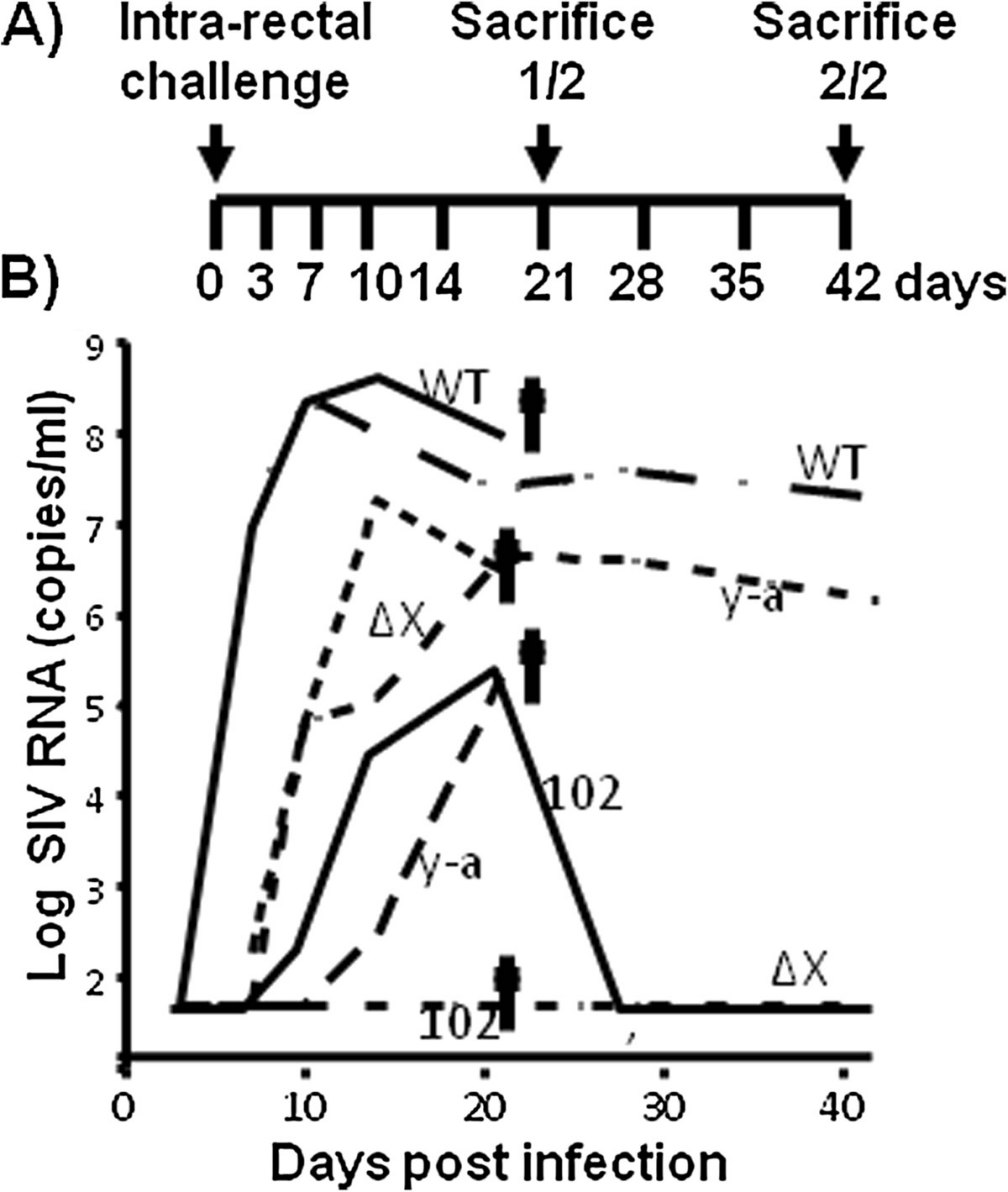
**Effect of Vpx mutations on SIV replication in pigtail macaques**. **A) **The design of the macaque infection shows that animals were inoculated by intra-rectal challenge, and one of two animals were sacrificed at 21 days after infection (✝), and the second animal in each group at 42 days after infection. Time points at which blood samples were obtained are also indicated. **B) **Plasma levels of SIV RNA (copies/ml) are indicated on a log scale for each of the 8 animals. The limit of detection in this assay was 50 copies/ml.

In order to assess the role of Vpx *in vivo*, wild-type SIVmne027 and the three Vpx mutant SIVmne viruses were used to infect juvenile pigtail macaque monkeys by the intra-rectal route of inoculation. For this purpose two animals per virus were infected with 10,000 TCID_50 _units of each virus. The experimental design is shown in Figure 9A. One animal in each group was sacrificed at 21 days post infection and the second animal on day 42. Blood was obtained at 0, 3, 7, 10, 14, 21, 28, 35, and 42 days after inoculation and used for virus load measurements (Figure 6B), sequence analysis, and CD4 assays. At necropsy, tissues were obtained from the rectum, colon, cecum, ileoceccal junction, ileum, jejunum, mesenteric lymph node, spleen, and a peripheral lymph node.

CD4 levels were depressed compared to baseline values at 10 days post infection in both wild-type SIVmne027 infected monkeys (by 9.6% and 60%, respectively, Table 1). In contrast all six macaques infected with the Vpx mutant SIVs showed no reduction in CD4 counts at 10 days post infection (CD4 increased by 3.1-79%, mean 31%, t < 0.03 compared to wild-type animals). Plasma virus load levels from SIVmne027 infected macaques reached peak values of 2.0 and 2.3 × 10^8 ^copies/ml at 7 and 10 days after inoculation, respectively (Figure 8, Table 1). In contrast, substantially lower peak viral loads were seen in animals infected with the mutant Vpx SIVs: SIVΔVpx infected macaques = < 50 and 3.0 × 10^6 ^copies/ml; SIV-X(y-a) infected macaques = 0.2 × 10^6 ^and 4.4 × 10^6 ^copies/ml; and SIV-X(102) infected macaques = < 50 and 0.3 × 10^6 ^copies/ml. To determine whether the levels of plasma viremia observed in the Vpx mutants resulted from reversion of the introduced mutations, sequence analyses were performed. Viral RNA was isolated from day 21 and 42 plasma; the *vpx *region was amplified by RT PCR, cloned into a plasmid vector, and individual clones were sequenced (data not shown). No clones were obtained from the plasma of both SIV-X(102) infected animals and the SIVΔVpx infected animal with undetectable levels of virus. Of the analyzed clones, neither SIVΔVpx nor SIV-X(y-a) showed reversions to wild-type sequence, indicating that the observed levels of replication of the Vpx mutants were not due to a reversion to wild-type sequences. Only one mutation was observed- a conserved mutation of A71V in the day 21 SIV-X(y-a) infected animal.

**Table 1:** *In situ *RNA hybridization (ISH) results from tissues of SIVmne infected pigtail macaques

SIVmne virus	Vpx-WT		ΔVpx		Vpx: y-a		Vpx: 102	
Macaque No.	27617	27614	27616	27613	27618	27615	28487	28486

Virus Loads								

Peak VL (x 10-6 copies/ml)	230	200	3.1	0	0.2	4.4	0.3	0

VL at Necropsy (x 10-6 copies/ml)	93.5	19.9	3.1	0	0.2	1.5	0	0

CD4 counts								

day 0	1057	2053	1277	2179	1823	1511	819	1176

day 10	955	808	2284	2615	2302	1802	1139	1212

day 21	1109	2740	1531	2818	1954	1701	460	869

day 42		1280		3547		1189	864	

ISH in Tissue								

Rectum	+ (LP, GALT)	ND	+	0	0	0	0	0

Colon	+ (LP, GALT)	ND	+ (GALT)	0	0	0	0	0

Cecum	+ (LP, GALT)	ND	+ (GALT)	0	0	0	0	0

Ileoceccal Junction	+ (LP, GALT)	ND	+ (GALT/LP)	0	+ (GALT)	+ (GALT)	0	0

Ileum	+	ND	0	0	+ (GALT)	0	0	0

Jejunum	+ (LP, GALT)	ND	+ (GALT)	0	0	0	0	0

Mesenteric Node	ND	+	+	0	0	0	0	0

Spleen	+	ND	+ (and LN)	0	0	+	0	0

Peripheral Node	+	ND	+	+	0	+	0	0

ISH was scored as +, SIV RNA expressing cells present; 0, SIV RNA expressing cell not present; ND, not done

*Abbreviations*: *GALT*, gut associated lymphoid tissue; *LN*, lymph node; *LP*, lamina propria;

### Pathology of SIVmne Vpx mutant infections

**Figure 10:**
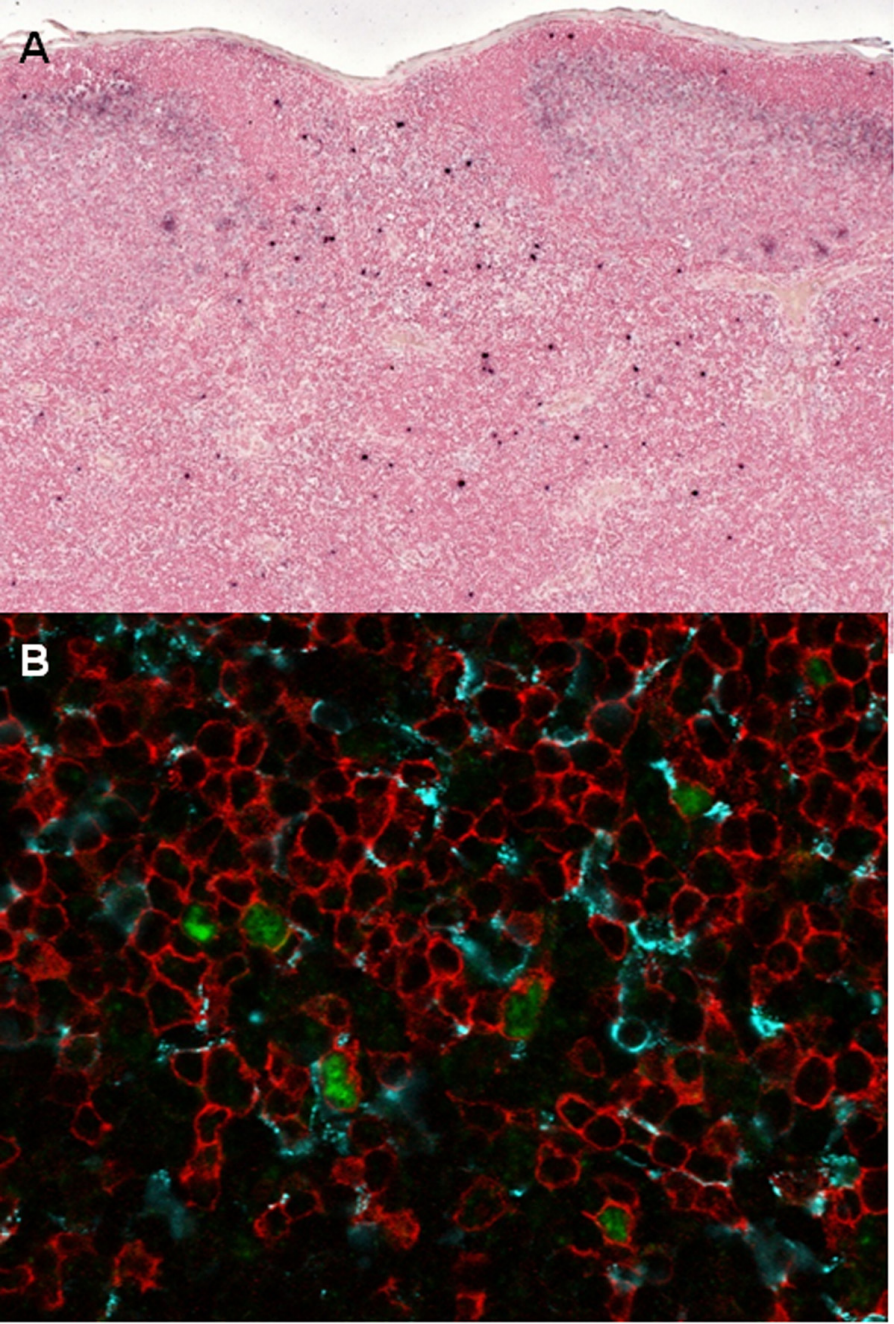
**In situ hybridization results of mesenteric node from a SIV infected pigtail macaque**. **A) **Mesenteric lymph node from a macaque 21 days after infection with wild type SIVmne contains SIV viral RNA expressing cells (dark blue). Magnification, 20×. **B) **Confocal microscopy (3 channel overlay) from a day 21 mesenteric lymph node from a wild type SIVmne infected macaque: ISH for SIV viral RNA was performed with a fluorescent tyramide signal amplification technique (green) and was combined with fluorescent IHC detection of HAM56-positive macrophages (blue) and CD3 positive lymphocytes (red). Magnification, 63×

To identify and type the SIV-expressing cells in lymph nodes and the gastrointestinal tract of the SIVmne027 infected animals, triple-label confocal microscopy using SIV-specific ISH (green), and immunohistochemistry for CD3+ T cells (red) and HAM56+ macrophages (blue) was performed (Figure 10). Quantification of SIV-infected cells showed that greater than 98% of the SIV + cells were T-cells. Virus was not found in dendritic cells.

To characterize the dissemination of the Vpx mutant viruses ISH for SIV was performed on the GI tract [gut-associated lymphoid tissue (GALT) and lamina propria (LP)], spleen, and mesenteric and peripheral nodes from all infected macaques. SIV positive cells were detected in all tissues of the wild-type SIVmne027 infected animal (Figure 10, Table 1). SIV positive cells were detected in a peripheral node of both of the SIVΔVpx infected macaques; however, virus was only detected in other tissues in the SIVΔVpx macaque (27616) exhibiting the detectable virus load. In this animal, virus was observed in all tissues but the ileum. SIV positive cells were detected only sporadically in the SIV-X(y-a) infected animals. RNA was found in the GALT of the ileocecal junction of both SIV-X(y-a) infected macaques, in the GALT in the ileum of macaque 27618, and the spleen and peripheral node of the other macaque (Table 1). Otherwise, tissues from SIV-X(y-a) infected macaques were negative by ISH for SIV RNA. The tissues from SIV-X(102) infected macaques did not contain any SIV positive cells by ISH assay.

## Discussion

The current studies confirmed and extended previous findings on the role of Vpx in SIV infection *in vitro *and dissemination *in vivo *[9,11,18,19,26]. Three SIV *vpx *mutants were examined in the current study (Figure 1). The unique positioning of vpx within the SIV genome allowed the mutagenesis without disrupting the ORFs of other viral genes. One SIV mutant eliminated Vpx expression (SIVΔVpx); the second mutant truncated Vpx and removed the C-terminal proline-rich domain (SIV-X102); and the third mutant contained a substitution of tyrosines 66, 69, and 71 with alanines [SIV-X(y-a)]. These three mutants were previously shown in HIV-2 to produce viruses with defects in replication in quiescent lymphocytes and MDMs [10,11]. The viruses were found to be defective in PIC nuclear import in quiescent lymphoid cells in culture. Moreover, the C-terminal truncation and tyrosine mutant also disrupted nuclear targeting of GFP-Vpx fusion proteins [10-12]. One caveat is that we cannot exclude the possibility that the C-terminal truncation and tyrosine mutations in the context of HIV-2 Vpx may have affected Vpx packaging into virus particles.

Not surprisingly, the SIV *vpx *mutants produced phenotypes similar to their HIV-2 counterparts. Each mutant was capable of generating high levels of virus equivalent to that of wild type SIV in proliferating CEMx174 cells and primary macaque PBMCs (Figures 3 and 5). The wild type SIVmne027 virus replicated to high levels in both human and macaque MDMs in culture, whereas all of the SIV *vpx *mutants were defective for replication in macrophages (Figures 4 and 5). Consistent with recent findings for a role of SIV Vpx in reverse transcription in macrophages, PCR analyses demonstrated that early reverse transcriptase products were decreased in macrophages infected with the SIV *vpx *mutant viruses compared to wild type SIVmne027 (Figure 6). As expected, and consistent with the reductions in early reverse transcription products, the levels of late reverse transcriptase products and integrated DNA, examined 24 and 48 hrs after infection, were also more profoundly decreased in the *vpx *mutant SIV infections compared to wild type SIVmne027 infected cells (Figure 6) [18,19]. Furthermore, reductions in reverse transcription by the Vpx mutants did not correlate with binding to DCAF1. It is notable however, that the current findings do not exclude additional effects of Vpx in macrophages in regulating steps before the initiation, or after the completion of reverse transcription, such as virus uncoating and preintegration complex nuclear import.

The *in vivo *studies with these SIV clones were performed in an established pigtail macaque infection model [30,31]. The number of experimental animals was limited by funding considerations to two macaques per virus. Unfortunately, this did not permit statistical analyses. Intra-rectal inoculation was chosen for the route of infection as it requires virus transport across a mucosal barrier, similar to that occurring during HIV infection in humans. This route of infection of macaques has been shown to reproducibly yield rapid virus dissemination to lymphoid tissues [26,32]. As expected, both wild type SIVmne027 infected macaques exhibited high levels of plasma virus load 7-10 days after infection. However, despite the consistency of the model, variation was seen in plasma virus loads among macaques infected with SIV *vpx *mutant viruses. All vpx mutant SIV infected animals had substantially lower peak virus loads than the SIVmne027 infected animals. Two of the six macaques infected with *vpx *mutant viruses failed to produce detectable virus loads in plasma. Another two produced peak levels of less than or equal to 300,000 copies/ml, which represented a > 460,000-fold reduction in virus load versus the SIVmne027 infected animals. The final two macaques infected with vpx mutant viruses had at least a 45-fold lower peak level of viremia compared to the SIVmne027 infected macaques. The overall trend of the *vpx *mutant viruses is consistent with previous findings that Vpx is required for dissemination *in vivo *[26].

An earlier study of SIVmac demonstrated progression to AIDS in the absence of a gene for *vpx *[33]. In this study, macaques were infected intravenously, unlike our study in which mucosal infection was utilized. It is notable that in the previous study, the number of infected lymphocytes was lower in animals infected with mutant compared to wild type SIV at all time points. Moreover, fewer positive cells were detected at 2 weeks by *in situ *hybridization in the lymph nodes in animals infected with mutant compared to wild type SIV. In addition, the time to death was longer in the animals infected with mutant compared to wild type SIV.

Our *in situ h*ybridization studies demonstrated SIV RNA expressing cells in both the mesenteric nodes of wild type SIVmne027 infected macaques and all other tissues examined, including rectal, intestinal epithelium, spleen, and peripheral node. These studies also confirmed previous observations that the predominant infected cell type is the intraepithelial lymphocyte rather than macrophages or dendritic cells [26,34]. In contrast, SIV RNA was found rarely in tissues and not identified in the rectum in five of the six macaques infected with SIV *vpx *mutant viruses. These findings confirm the role of Vpx in dissemination of virus, and extend findings obtained with SIVsmm pbj to a second virus, SIVmne027 [26]. More importantly, these findings demonstrate that specific mutations within Vpx that disrupt replication *in vitro *also inhibit virus dissemination *in vivo*. It is likely that these functions of Vpx are important for infection of resting T cells in the rectum [35]. Perhaps nucleotide triphosphate levels for efficient reverse transcription are limiting in quiescent T cells, similar to that in myeloid cells, which could explain the requirement for Vpx for efficient SIV infection [36,37]

One notable difference of the current results compared with those reported with SIVsmm pbj infection report is that in the current study Vpx also appears to be important for amplification of local infection prior to distant dissemination [26]. This was demonstrated by the failure to identify SIV RNA in the rectal crypts and lamina propria within five of the six Vpx mutant SIV-infected animals. It is possible that differences in experimental design contributed to this distinct finding. A striking difference between these studies was the use of acutely lethal strain of SIVsmm pbj in the previous study, and a less rapidly lethal AIDS-inducing strain of SIVmne in the current study. Other technical differences between these studies may also account for this result, possibly related to the dose of virus used in the inoculums (10,000 TCID_50 _in the current study compared to 2,000 TCID_50 _previously), or the fact that in the current study tissues were examined at 21 and 42 days after infection, whereas in the previous study, *in situ *hybridization was performed at earlier time points. Despite these caveats, both studies demonstrate an important role for Vpx in establishing SIV infection *in vivo*. Moreover, the current study demonstrates a correlation of the ability of Vpx to overcome the restriction to virus replication in monocyte-derived macrophages *in vitro *and successful establishment of virus infection *in vivo*.

## Conclusions

The current study utilized SIVmne027 with a mutation that prevents Vpx expression, or mutations in the conserved tyrosines or the C-terminal proline-rich domain that inhibit the nuclear import of Vpx. All three mutant forms of Vpx attenuated SIV replication in monocyte-derived macrophages, reduced the synthesis and integration of viral DNA, and were impaired in the ability to promote SAMHD1 degradation. Moreover, all the Vpx mutants were defective in promoting efficient replication of SIVmne in pigtail macaques, and were associated with decreased viral RNA in gut-associated lymphoid tissue and lamina propria, spleen, and mesenteric nodes. These findings demonstrate that the ability of Vpx to overcome restrictions in culture is also likely to be important for establishing infection *in vivo*.

## Methods

### Viruses

The infectious, pathogenic clone, SIVmne027 (SIVmne), was obtained from the lymph node of a macaque with a declining CD4 cell count and early signs of AIDS [29]. Mutations in *vpx *were constructed by splice overlap PCR mutagenesis, engineered into the SIVmne027 molecular clone, and confirmed by restriction enzyme digests and sequence analysis. For *in vitro *replication assays, molecular clones were transfected into 293T cells using TransIT-LT (Mirus Bio), virus harvested 48-72 hrs later, and quantified by SIV p27 antigen ELISA (ZeptoMetrix). For PCR assays, SIV molecular clones were co-transfected with a VSV-G expression clone, and quantified by JC53 assays [38,39]. For *in vivo *replication studies, SIV stocks were grown in CEMx174 cells after infection with filtered SIV virus stocks generated from 293T cells. Supernatants were harvested 7-10 days post-inoculation, passed through 0.45 mM syringe filters, and stored at -80°C until needed. Infectious virus was quantified using the sMAGI assay [40].

### *In *vitro replication assays

CEM and CEM × 174 cells were grown in RPMI-1640 with 10% fetal calf serum, 4 mM L-glutamine, 1 mM sodium pyruvate, and 100 μg/ml penicillin-streptomycin. Human peripheral blood monocytes were obtained by leukopheresis from HIV-1 and hepatitis seronegative donors and purified by counter-current centrifugal elutriation to > 99% purity as assessed by immunolabeling with anti-CD68 [41]. Monocytes were plated for 7-14 days in RPMI-1640 supplemented with 500 U/ml M-CSF, and the supplements used above for CEM × 174 cells. Pigtailed macaque PBMCs were separated from whole blood by density centrifugation with 95% LSM (MP Biomedicals, Solon, OH) and stimulated with 2 μg/ml of PHA (Sigma-Aldrich, St. Louis, MO) and 10 ng/ml of human interleukin-2 (IL-2; R&D Systems, Minneapolis, MN) in RPMI 1640, supplemented with 10% heat-inactivated FCS, 4 mM glutamine, 100 U/ml of penicillin, and 100 μg/ml streptomycin for 3 days and then washed and maintained in complete RPMI 1640 supplemented with IL-2. Reverse transcriptase assays were performed as previously described [41].

Pig-tail monocytes were isolated by plastic adherence from pig-tailed macaque PBMCs and differentiated into macrophages (MDMs) as previously described [30]. MDMs were infected at a multiplicity of infection (MOI) of 0.01. Viral replication was monitored by measuring SIV p27 in culture supernatants by ELISA (Advanced Bioscience Laboratories, Inc., Kensington, MD).

For real time PCR assays, monocyte-derived macrophages were infected with 300 or 200 ng (p27) of virus, respectively, pretreated with TURBO DNase (Ambion, Austin, TX). DNA was harvested 24 and 48 hrs after infection with the DNeasy tissue kit (Qiagen). For early reverse transcriptase products, the following primers in U5 were used: 5'GAGGCTGGCAGATTGAGCCCTG and 5'GGTCCTAACAGACCAGGGTCT. For late reverse transcriptase products, the following primers in *gag *were used: 5'TCCGTCTTGTCAGGGAAGAAAGCA and 5'AGCCTGTTGGCACTAATGGAGCTA. Alu-integration assays were performed by real time PCR as previously described, except that the primer in the 2^nd ^round PCR complementary to SIVmne LTR was 5'GGTCCTAACAGACCAGGGTCT [13]. For total viral DNA assays, AZT treatment was performed to measure background levels, which were subtracted from experimental values. For Alu-integration assays, a control without Alu primers was performed, and background levels were subtracted from experimental values.

### Packaging

Plasmids encoding FLAG-tagged SIV Vpx mutants and Wt Vpx (3 μg), in conjunction with proviral plasmids encoding SIVmne lacking Vpx expression (3 μg), were transiently co-transfected into 293 T cells using *Trans*IT-LT1 Reagent (Mirus Bio). MG-132 (5 μM) was added to all cells 24 h post-transfection. After 48 hours, supernatants were collected and filtered through 0.45 micron filters while the cells were collected and lysed in Laemmli sample buffer. Virions were ultracentrifuged on a 20% sucrose cushion for 1 h, at 45,000 rpm, and in an SW55 Beckman rotor. Virion samples were resolved on PAGE-SDS, transferred to a PVDF, and blotted with Flag mAb (Sigma M2) for Vpx and HIV-1 Gag serum for Capsid.

### Immunoprecipitation

Plasmids encoding FLAG-tagged SIV Vpx mutants and Wt Vpx (4 μg) were transiently transfected into 293 T cells *Trans*IT-LT1 Reagent (Mirus Bio). MG-132 (5 μM) was added to all cells 24 h post-transfection. After 48 h, cells were lysed using Triton X-100 detergent-containing buffer (10 mM Tris-HCl pH7.5, 150 mM NaCl, 1% Triton X-100, and complete protease inhibitor (Roche)). After clarification, 5% of total cell lysate was saved for Vpx and DCAF1 blotting, the rest of the lysate was used for Vpx pulldown with mouse anti-FLAG antibody (Sigma) and protein A/G PLUS-agarose (Santa Cruz Biotechnology). Agarose beads were washed 5 times with 1XPBS. Vpx was eluted from agarose with SDS-PAGE sample buffer, boiled, and resolved using 8% SDS-PAGE. Vpx was detected using mouse anti-FLAG (Sigma) whereas DCAF1 was detected using rabbit anti-VprBP (ProteinTech Group) and HRP-linked anti-mouse (Sigma) and anti-rabbit (Thermo Scientific) secondary antibodies. Bands were developed with SuperSignal West Femto Substrate (Thermo Scientific) and developed using Bio-Rad ChemiDoc XRS^+ ^(Bio-Rad).

Increasing amounts of plasmids encoding FLAG-tagged SIV Vpx mutants (0.0 μg, 0.4 μg, 0.8 μg, and 1.2 μg for Wt Vpx and Y-A Vpx, and 0.0 μg, 1.2 μg, 1.6 μg, and 2.0 μg for 102 Vpx) and FLAG-tagged human SAMHD1 (0.1 μg) were transiently transfected into 293 T cells with TransIT-LT1 Reagent (Mirus Bio). After 72 hrs, whole-cell extracts were prepared with Laemmli sample buffer and samples were analyzed on PAGE-SDS. PVDF-bound proteins were incubated with anti-Flag (Sigma M2) and anti-Actin-HRP (Santa Cruz Biotechnology) antibody. The intensity of bands was determined with image analysis software (Bio-Rad Image Lab 3.0). Densitometry values were normalized to actin and set relative to SAMHD1 in the absence of Vpx. A 2-tailed t-test was used to assess the different results.

### *In vivo *infection assays

Juvenile pig-tailed macaques (*M. nemestrina*), 2 yrs old and negative for SIV and simian type D retrovirus, were inoculated intrarectally with equal amounts (10^4 ^tissue culture infectious doses, TCID_50_, as determined by a MAGI assay) of SIVMne027 [40]. From each inoculated macaque serial samples of peripheral blood and plasma were obtained at days 0, 3, 7, 10, and 14, and then weekly to monitor CD4+ T lymphocyte levels, and viral loads. All animals were maintained and cared for in accordance with the guidelines of the AAALAC and the Animal Care and Use Committee of the Southwest National Primate Research Center at The Texas Biomedical Research Institute. Lymphocyte phenotyping assays were performed by flow cytometry with antibodies to CD3 (clone SP34.2), CD4 (clone L200), and CD8 (clone RPA-T8) (BD Biosciences, San Jose, CA). SIV RNA plasma virus load assays were run by a nucleic acid based amplification assay (Advances Biosciences).

### *In situ *hybridization (ISH) assays

Formalin-fixed, paraffin-embedded tissues were assayed for SIV expression by an ISH assay [42]. Sections from the widest part of the lymph node or GI tract were used for staining. Sections were hybridized overnight with a full-length sense or antisense SIVmac239 digoxigenin-UTP labeled riboprobe, followed by sheep anti-digoxigenin-alkaline phosphatase (Roche Molecular) and nitroblue tetrazolium-5-bromo-4-chloro-3-indoyl-β-D-galactopyranoside (NBT/BCIP). The slides were counter stained with nuclear fast red and photographed with a Zeiss Axiophot microscope. The number of SIV + cells observed in different sections was very consistent from slide to slide. For confocal microscopy, tissue sections, hybridized as described above, were incubated with sheep anti-digoxigenin-horseradish peroxidase, and detected with a fluorescein tyramide signal amplification technique [42]. The sections were stained with HAM56, anti-human macrophage antibody (DAKO) followed by a biotinylated secondary and streptavidin-Alexa633 (Molecular Probes). The samples were then incubated with rabbit anti-human CD3 (DAKO), followed by goat anti-rabbit IgG-Alexa594 (Molecular Probes) and cover slipped with Vectashield Hardset (Vector Laboratories). Triple stained sections were photographed with a Leica confocal scanning microscope.

## Competing interests

The authors declare that they have no competing interests.

## Authors' contributions

MB, JTK, and LR made substantial contributions to conception and design, MB, JTK, CB, XC, AM, AL, VH, RT, LG, and VH made contribution to data acquisition, MB, JTK, AM, CB, VH, and LR made contributions to data analysis, MB, JTK, CB, XC, VH, and LR have been involved with drafting the manuscript. All authors read and approved the final manuscript.

## Supplementary Material

Additional file 1 **Figure S1 Expression of SIV Vpx mutants**. SIV molecular clones lacking Vpx expression were complemented *in trans *with indicated Vpx mutants and Vpx WT by transient transfection of 293T cells. MG132 was added 24 h post-transfection. Cells were lysed in Laemmli sample buffer at 48 h and Vpx expression were analyzed by immunoblot using FLAG mAb. (PDF 175 kb). Click here for file

## References

[B1] Sharp PM Bailes E Stevenson M Emerman M Hahn BH Gene acquisition in HIV and SIV Nature 1996 383 586 587 10.1038/383586a0 8857532 PMC9514223

[B2] Tristem M Marshall C Karpas A Petrik J Hill F Origin of vpx in lentiviruses Nature 1990 347 341 342 2145513 10.1038/347341b0

[B3] Tristem M Purvis A Quicke DLJ Complex evolutionary history of primate lentiviral vpr genes Virology 1998 240 232 237 10.1006/viro.1997.8929 9454696

[B4] Cohen EA Subbramanian RA Gottlinger HG Role of auxiliary proteins in retroviral morphogenesis Curr Top Microbiol Immunol 1996 214 219 235 10.1007/978-3-642-80145-7_7 8791729

[B5] Kappes JC Viral protein X Curr Top Microbiol Immunol 1995 193 121 132 10.1007/978-3-642-78929-8_7 7648872

[B6] Trono D When accessories turn out to be essential Nat Med 1998 4 1368 1369 10.1038/3953 9846571

[B7] Mahnke LA Belshan M Ratner L Analysis of HIV-2 Vpx by modeling and insertional mutagenesis Virology 2006 348 165 174 10.1016/j.virol.2005.12.023 16457868

[B8] Pancio H Ratner L Human immunodeficiency virus 2 Vpx-Gag interaction J Virol 1998 72 5271 5275 9573303 10.1128/jvi.72.6.5271-5275.1998 PMC110118

[B9] Fletcher TM Brichacek B Sharova N Newman MA Stivathis G Sharp PM Emerman M Hahn BH Stevenson M Nuclear import and cell cycle arrest functions of the HIV-1 Vpr protein are encoded by two separate genes in HIV-2/SIVsm EMBO J 1996 15 6155 6165 8947037 PMC452436

[B10] Pancio H Heyden NV Ratner L The C-terminal proline-rich tail of HIV-2 Vpx is necessary for nuclear localization of the viral preintegration complex in nondividing cells J Virol 2000 74 6162 6167 10.1128/JVI.74.13.6162-6167.2000 10846100 PMC112115

[B11] Belshan M Mahnke LA Ratner L Conserved amino acids of the human immunodeficiency virus type 2 Vpx nuclear localization signal are critical for nuclear targeting of the viral preintegration complex in non-dividing cells Virology 2006 346 118 126 10.1016/j.virol.2005.10.036 16325220

[B12] Belshan M Ratner L Identification of the nuclear localization signal of human immunodeficiency virus type 2 Vpx Virology 2003 311 7 15 10.1016/S0042-6822(03)00093-X 12832198

[B13] Cheng X Ratner L Hsp40 facilitates nuclear import of the human immunodeficiency virus type 2 Vpx-mediated preintegration complex J Virol 2008 82 1229 1237 10.1128/JVI.00540-07 18032501 PMC2224430

[B14] Mueller SM Jung R Weiler S Lang SM Vpx proteins of SIVmac239 and HIV-2ROD interact with the cytoskeletal protein alpha-actinin 1 J Gen Virol 2004 85 3291 3203 10.1099/vir.0.80198-0 15483243

[B15] Singhal PK Kumar PR Rao MRKS Kyasani M Mahalingam S Simian imunodeficiency virus Vpx is imported into the nucleus via importin alpha-dependent and -independent pathways J Virol 2006 80 526 536 10.1128/JVI.80.1.526-536.2006 16352576 PMC1317556

[B16] Vodicka MA Koepp DM Silver PA Emerman M HIV-1 Vpr interacts with the nuclear transport pathway to promote macrophage infection Genes Dev 1998 12 175 185 10.1101/gad.12.2.175 9436978 PMC316441

[B17] Goujon C Arfi V Pertel T Luban J Lienard J Rigal D Darlix JL Cimarelli A Characterization of simian immunodeficiency virus SIVsm/human immunodeficiency virus type 2 Vpx function in human myeloid cells J Virol 2008 82 12335 12345 10.1128/JVI.01181-08 18829761 PMC2593360

[B18] Sharova N Wu Y Zhu X Stranska R Kaushik R Sharkey M Stevenson M Primate lentiviral Vpx commandeers DDB1 to counteract a macrophage restriction PLoS Pathog 2008 4 e1000057 10.1371/journal.ppat.1000057 18451984 PMC2323106

[B19] Srivastava S Swanson SK Manel N Florens L Washburn MP Skowronski J Lentiviral Vpx accessory factor targets VprBP/DCAF1 substrate adaptor for cullin 4 E3 ubiquitin ligase to enable macrophage infection PLoS Pathog 2008 4 e100059 10.1371/journal.ppat.1000059 PMC2330158 18464893

[B20] Fujita M Otsuka M Miyoshi M Khamsri B Nomaguchi M Adachi A Vpx is critical for reverse transcription of the human immunodeficiency virus type 2 genome in macrophages J Virol 2008 82 7752 7756 10.1128/JVI.01003-07 18495778 PMC2493314

[B21] LeRouzic E Belaidouni N Estrabaud E Morel M Rain JC Transy C Margottin-Goguet F HIV1 Vpr arrests the cell cycle by recruiting DCAF1/VprBP, a receptor of the Cul4-DDB1 ubiquitin ligase Cell Cycle 2007 15 182 188 10.4161/cc.6.2.3732 17314515

[B22] Hrecka K Hao C Gierszewska M Swanson SK Kesik-Brodacka M Srivastava S Florens L Washburn MP Skowronski J Vpx relieves inhibition of HIV-1 infection of macrophages mediated by the SamHD1 protein Nature 2011 474 658 661 10.1038/nature10195 21720370 PMC3179858

[B23] Laguette N Sobhian B Casartelli N Ringeard M Chable-Bessia C Segeral E Yatim A Emiliani S Schwartz O Benkirane M SAMHD1 is the dendritic- and myeloid-cell-specific HIV-1 restriction factor counteracted by Vpx Nature 2011 474 654 657 10.1038/nature10117 21613998 PMC3595993

[B24] Goldstone DC Ennis-Adeniran V Hedden JJ Groom HC Rice GI Christodoulou E Walker PA Kelly G Haire LF Yap MW HIV-1 restriction factor SAMHD1 is a deoxynucleoside triphosphate triphosphohydrolase Nature 2011 480 379 382 10.1038/nature10623 22056990

[B25] Powell RD Holland PJ Hollis T Perriono FW The Aicardi-Goutieres syndrome gene and HIV-1 restriction factor SAMHD1 is a dGTP-regulated deoxynucleotide triphosphohydrolase Journal of Biological Chemistry 2011 286 43596 43600 10.1074/jbc.C111.317628 22069334 PMC3243528

[B26] Hirsch VM Sharkey ME Brown CR Brichacek B Goldstein S Wakefield J Byrum R Elkins BH Lifson JD Stevenson M Vpx is required for dissemination and pathogenesis of SIVsmPBj: evidence of macrophage-dependent viral amplification Nat Med 1998 4 1401 1408 10.1038/3992 9846578 PMC9513717

[B27] Kimata JT Kuller L Anderson DB Dailey P Overbaugh J Emerging cytopathic and antigenic simian immunodeficiency virus variants influence AIDS progression Nat Med 1999 5 535 541 10.1038/8414 10229230

[B28] Patel PG YuKimata MT Biggins JE Wilson JM Kimata JT Highly pathogenic simian immunodeficiency virus mne variants that emerge during the course of infection evolve enhanced infectivity and the ability to downregulate CD4 but not class I major histocompatibility complex antigens J Virol 2002 76 6425 6434 10.1128/JVI.76.13.6425-6434.2002 12050354 PMC136284

[B29] Kimata JT Mozaffarian A Overbaugh J A lymph node-derived cytopathic simian immunodeficiency virus Mne variant replicates in non-stimulated peripheral blood mononuclear cells J Virol 1998 72 245 256 9420221 10.1128/jvi.72.1.245-256.1998 PMC109370

[B30] Biesinger T White R Kimata-Yu MT Wilson BK Allan JS Kimata JT Relative replication capacity of phenotypic SIV variants during primary infections differs with route of inoculation Retrovirology 2010 7 88 10.1186/1742-4690-7-88 20942954 PMC2964591

[B31] Kuller L Thompson J Watanabe R Iskandriati D Alpers CE Morton WR Agy MB Mucosal antibody expression following rapid SIV(Mne) dissemination in intrarectally infected Macaca nemestrina AIDS Res Hum Retroviruses 1998 14 1345 1356 10.1089/aid.1998.14.1345 9788676

[B32] Coudedel-Courteille A Butor C Jillard V Guillet J-G Venet A Dissemination of SIV after rectal infection preferentially involves paracolic germinal centers Virology 1999 260 277 294 10.1006/viro.1999.9809 10417263

[B33] Gibbs JS Lackner AA Lang SM Simon MA Sehgal PK Daniel MD Desrosiers RC Progression to AIDS in the absence of a gene for vpr or vpx J Virol 1995 69 2378 2383 7884883 10.1128/jvi.69.4.2378-2383.1995 PMC188910

[B34] Li Q Duan L Estes JD Ma Z-M Rourke T Wang Y Reilly C Carlis J Miller CJ Haase AT Peak SIV replication in resting memory CD4+ T cells depletes gut lamina propria CD4+ T cells Nature 2005 434 1148 1152 15793562 10.1038/nature03513

[B35] Zhang Z-Q Schuler T Zupancic M Wietgrefe S Staskus KA Reimann KA Reinhart TA Rogan M Cavert W Miller CJ Sexual Transmission and Propagation of SIV and HIV in Resting and Activated CD4^+ ^T Cells Science 1999 286 1353 1357 10.1126/science.286.5443.1353 10558989

[B36] Zack JA Arrigo SJ Weitsman SR Go AS Haislip A Chen ISY HIV-1 entry into quiescent primary lymphocytes: molecular analysis reveals a labile, latent viral structure Cell 1990 61 213 222 10.1016/0092-8674(90)90802-L 2331748

[B37] O'Brien WA Namazi A Kalhor H Mao SH Zack JA Chen IS Kinetics of human immunodeficiency virus type 1 reverse transcription in blood mononuclear phagocytes is slowed by limitations of nucleotide precursors J Virol 1994 68 1258 1263 7507180 10.1128/jvi.68.2.1258-1263.1994 PMC236573

[B38] McCulley A Morrow CD Complementation of human immunodeficiency virus type 1 replication by intracellular selection of Escherichia coli formula supplied in trans J Virol 2006 80 9641 9650 10.1128/JVI.00709-06 16973568 PMC1617247

[B39] Ory DS Neugeboren BA Mulligan RC A stable human-derived packaging cell line for production of high titer retrovirus/vesicular stomatitis virus G pseudotypes Proc Natl Acad Sci 1996 93 11400 11406 10.1073/pnas.93.21.11400 8876147 PMC38069

[B40] Chackerian B Haigwood NL Overbaugh J Characterization of a CD4-expressing macaque cell line that can detect virus after a single replication cycle and can be infected by diverse simian immunodeficiency virus isolates Virology 1995 213 386 394 10.1006/viro.1995.0011 7491763

[B41] Westervelt P Trowbridge DB Epstein LG Blumberg BM Li Y Hahn BH Shaw GM Price RW Ratner L Macrophage tropism determinant of human immunodeficiency virus type 1 in vivo J Virol 1992 66 2577 2582 1548783 10.1128/jvi.66.4.2577-2582.1992 PMC289061

[B42] Brown CR Czapiga M Kabat J Dang Q Ourmanov I Nishimura Y Martin MA Hirsch VM Unique pathology in simian immunodeficiency virus-infected rapid progressor macaques is consistent with a pathogenesis distinct from that of classical AIDS J Virol 2007 81 5594 5606 10.1128/JVI.00202-07 17376901 PMC1900277

