# Supplementary material for: Vpx is Critical for SIVmne infection of pigtail macaques
Source: Retrovirology. 2012 Apr 24;9:32. doi: 10.1186/1742-4690-9-32 (PMC3353869; doi:10.1186/1742-4690-9-32)
Supplement: Additional file 1 — Figure S1 Expression of SIV Vpx mutants. SIV molecular clones lacking Vpx expression were complemented in trans with indicated Vpx mutants and Vpx WT by transient transfection of 293T cells. MG132 was added 24 h post-transfection. Cells were lysed in Laemmli sample buffer at 48 h and Vpx expression were analyzed by immunoblot using FLAG mAb. (PDF 175 kb). [file 1742-4690-9-32-S1.PDF]

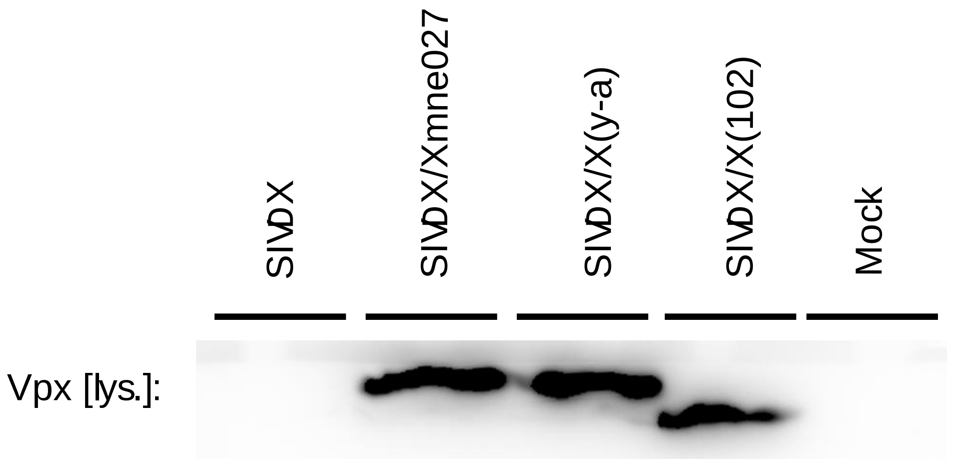

Supplementary Figure 1. Expression of SIV Vpx mutants. SIV molecular clones lacking *vpx* were complemented in *trans* with indicated Vpx mutants and wild type Vpx with Flag epitope tags by transient transfection of 293T cells. MG132 was added 24 h post-transfection. Cells were lysed in Laemmli sample buffer at 48 h and Vpx expression was analyzed by immunoblot using anti-Flag monoclonal antibody
